# RETRACTION: LncRNA NR2F1‐AS1 Regulates Hepatocellular Carcinoma Oxaliplatin Resistance by Targeting ABCC1 via miR‐363

**DOI:** 10.1111/jcmm.70908

**Published:** 2025-10-14

**RETRACTION**: H. Huang , J. Chen , C.‐M. Ding , X. Jin , Z.‐M. Jia and J. Peng , LncRNA NR2F1‐AS1 Regulates Hepatocellular Carcinoma Oxaliplatin Resistance by Targeting ABCC1 via miR‐363,” Journal of Cellular and Molecular Medicine 22, no. 6 (2018): 3238–3245, 10.1111/jcmm.13605.29602203 PMC5980138

The above article, published online on 30 March 2018 in Wiley Online Library (wileyonlinelibrary.com), has been retracted by agreement between the journal Editor‐in‐Chief, Stefan N. Constantinescu; the Foundation for Cellular and Molecular Medicine; and John Wiley & Sons Ltd. The retraction has been agreed upon following concerns raised by a third party. Following investigation, several elements from Figures 3A, 3C, and 5D were found to be duplicated in two previously published articles by different author groups. Furthermore, some of the images were shown as representing different scientific contexts. The authors cooperated with the investigation and provided some supporting data for evaluation. They explained that images from other journals had been collected during their study for reference purposes but were inadvertently included in their own article. As a result, the editors consider the results and conclusions invalid. The authors were informed of the decision to retract but did not respond to requests for comment.

